# Refractory abdominal compartment syndrome secondary to ruptured abdominal aortic aneurysm treated with total colectomy and upper rectal resection: A case report and literature review

**DOI:** 10.1097/MD.0000000000048285

**Published:** 2026-04-24

**Authors:** Yangyang Jiang, Jindi Ni, Lu Zhang, Lijing Jiang, Xiang Li

**Affiliations:** a Department of Critical Care Medicine, Minhang Hospital, Fudan University, Shanghai, China.

**Keywords:** abdominal compartment syndrome, multiple organ dysfunction syndrome, ruptured abdominal aortic aneurysm

## Abstract

**Rationale:**

Ruptured abdominal aortic aneurysm (RAAA) is a critical condition often complicated by multiple organ dysfunction syndrome (MODS), which can significantly impede recovery following surgical intervention. Abdominal compartment syndrome (ACS) is a widely recognized but highly lethal postoperative complication.

**Patient concerns:**

A 69-year-old male presented with acute abdominal pain and hemorrhagic shock.

**Diagnoses:**

RAAA with active extravasation was confirmed, and following surgical repair, the patient developed MODS, including hepatorenal failure and acute respiratory distress syndrome. Twenty-one days postoperatively, secondary ACS led to colonic ischemia.

**Interventions:**

The patient was managed using staged surgical interventions, including decompressive laparotomy, negative pressure wound therapy systems, and definitive total colectomy with upper rectal resection due to refractory ACS characterized by transmural colonic necrosis.

**Outcomes:**

The patient showed significant recovery and was discharged after 81 days of hospitalization. At 1-month follow-up, he reported normal oral intake and no recurrence of major complications.

**Lessons:**

This case illustrates the effectiveness of a phased damage control strategy in managing high-risk RAAA patients, demonstrating that early prediction, timely intervention, and multidisciplinary care can significantly improve survival outcomes.

## 1. Introduction

Abdominal aortic aneurysm (AAA) is a prevalent condition, affecting approximately 8% of the global population. It stands as a leading cause of mortality in the United States, the United Kingdom, and Europe, especially among individuals aged 60 and older. Research indicates that rupture of an abdominal aortic aneurysm (RAAA) accounts for between 53% and 65% of in-hospital deaths and 43% of deaths following intervention.^[1]^ While early detection and treatment of elective AAA have improved over the years, RAAA continues to be a major contributor to mortality. The advent of endovascular aortic repair (EVAR) has led to a higher number of elective procedures, enabling the treatment of asymptomatic AAA patients before rupture occurs. Despite this progress, the mortality associated with RAAA remains influenced by several factors, such as the timeliness of patient transfer, surgical suitability, perioperative complications, and the availability of clear treatment protocols in high-volume centers. Alarmingly, nearly two-thirds of RAAA patients die before even reaching the hospital, with another 20% succumbing before they can undergo surgery. Although advancements in anesthesia and intensive care have led to reduced intraoperative deaths, the overall 30-day mortality rate has not shown significant improvement.^[1]^ This lack of progress is often linked to the aging patient demographic and the prevalence of comorbidities, with about 50% of patients dying in the ICU due to postoperative complications that frequently lead to multi-organ failure.^[2]^ Hence, while analyzing large-scale data is essential, it is equally important to examine individual success stories that highlight effective management techniques. This case demonstrates the effectiveness of a structured, triple-phase damage control approach in mitigating the MODS-ACS cascade in complex RAAA management. By closely reviewing these successful rescue cases, we can identify key interventions that significantly improve patient outcomes. In turn, this detailed case review offers invaluable insights into refining treatment protocols, ensuring that we continue to enhance survival rates and quality of life for RAAA patients.

## 2. Patient information

A 69-year-old male smoker with a 40-pack-year history and uncontrolled hypertension (20 years, BP 140–170/90–110 mm Hg) presented without a significant family history of cardiovascular disease. His adherence to antihypertensive treatment was intermittent, and he denied significant alcohol consumption.

## 3. Clinical findings

The patient presented with acute abdominal pain lasting 10 hours, accompanied by hemodynamic instability: hypotension (65/44 mm Hg), tachycardia (130 bpm), and marginal oxygenation (SpO_2_ 92%). Admission labs revealed anemia (Hb 7.7 g/dL), leukocytosis (WBC 14.1 × 10^9^/L), thrombocytopenia (PLT 61 × 10^9^/L), and normal hepatorenal function (INR 1.12).

## 4. Timeline

Within 1 hour of admission, the patient underwent emergent Phase I surgery, including resuscitative endovascular balloon occlusion of the aorta (REBOA) followed by midline laparotomy and synthetic Dacron graft replacement. Postoperatively, the patient developed multiple organ dysfunction syndrome (MODS), including renal failure, hyperbilirubinemia, and acute respiratory distress syndrome (ARDS), necessitating continuous renal replacement therapy (CRRT) and molecular adsorbent recirculating system (MARS) for hepatic support. On postoperative day 21, secondary abdominal compartment syndrome (ACS) developed, leading to the need for decompressive laparotomy, diverting loop ileostomy, and negative pressure wound therapy (ABTHERA™). Despite some improvement, the patient developed colonic ischemia and fresh melena by day 28. A multidisciplinary decision led to a total colectomy with upper rectal resection. The patient showed significant recovery over 81 days in the hospital, with normalized renal and hepatic function, and was discharged ambulating independently with restored health. At a 1-month follow-up, he reported normal oral intake and intermittent loose stools, with no recurrence of complications.

## 5. Diagnostic assessment

**Figure 1.**
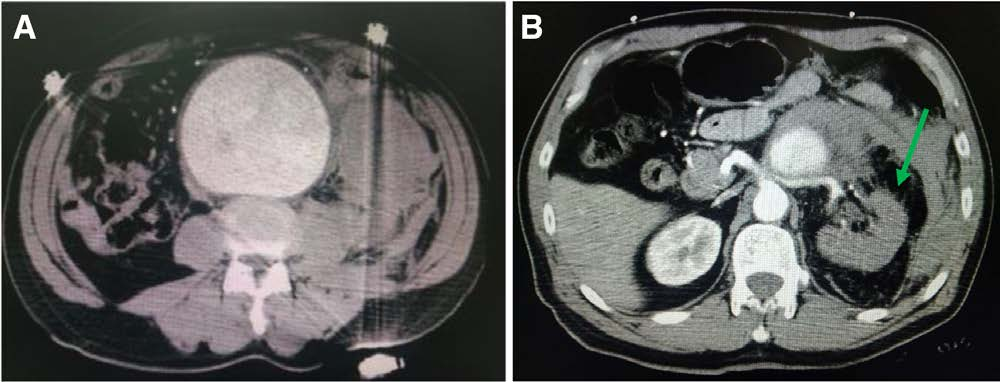
(A) Abdominal aortic aneurysmal dilation measuring 10.2  × 8.5  × 6 cm, with high-density attachment to the vessel wall. (B) Post-enhancement imaging reveals interrupted continuity of the left renal artery, with no significant enhancement observed in the left renal parenchyma.

Contrast-enhanced abdominal computed tomography angiography (CTA) confirmed the diagnosis of ruptured abdominal aortic aneurysm, showing: an infrarenal aortic aneurysm measuring 10.2 × 8.5 × 6 cm with mural thrombus (Fig. 1A); active contrast extravasation indicated by periaortic hyperdense collections (78 Hounsfield units) and peritoneal fluid layering; compromised left renal perfusion with arterial patency but parenchymal hypoenhancement and loss of cortico-medullary differentiation (Fig. 1B) and severe atherosclerotic dilation of the bilateral common iliac arteries (right: 18 mm, left: 16 mm).

## 6. Therapeutic intervention

Given the patient’s hemodynamic instability and radiologic confirmation of RAAA with active extravasation, an emergent Phase I damage control operation was initiated within 1 hour. Resuscitative endovascular balloon occlusion of the aorta (REBOA) was deployed via femoral access to transiently stabilize circulatory parameters. This was followed by midline laparotomy and resection of the aneurysmal segment, with interposition of a synthetic Dacron graft. Intraoperatively, estimated 3,850mL of intraperitoneal blood was evacuated. Hemostatic resuscitation included transfusion of 8 units of packed red blood cells, 1200 mL of fresh frozen plasma, and 1100 mL of autologous blood. The patient was transferred postoperatively to the ICU under vasopressor support and mechanical ventilation.

Within 24 hours, the patient progressed to full-blown MODS, exhibiting oliguric renal failure, hyperbilirubinemia, and severe ARDS. CRRT was commenced, along with molecular adsorbent recirculating system therapy for hepatic support. Mechanical ventilation settings were optimized using lung-protective strategies: low tidal volume (Vt 4–6 mL/kg), PEEP 14 cmH_2_O with PaO_2_/FiO_2_ ratio 68.2, and permissive hypercapnia. Bronchoalveolar lavage with next-generation sequencing (NGS) was performed to rule out opportunistic infections. Hemodynamic parameters were monitored using Pulse Contour Cardiac Output Monitoring (PICCO) technology to guide fluid management. Sedation was maintained using remifentanil, midazolam, and dexmedetomidine. Despite these interventions, the patient developed progressive abdominal distension, vomiting, and loss of bowel sounds on postoperative day 21. Intra-abdominal pressure (IAP) measured 24 mm Hg via bladder transducer, confirming abdominal compartment syndrome (IAP > 20 mm Hg with new-onset renal and respiratory failure), per the World Society of the Abdominal Compartment Syndrome (WSACS) consensus definitions.

**Figure 2.**
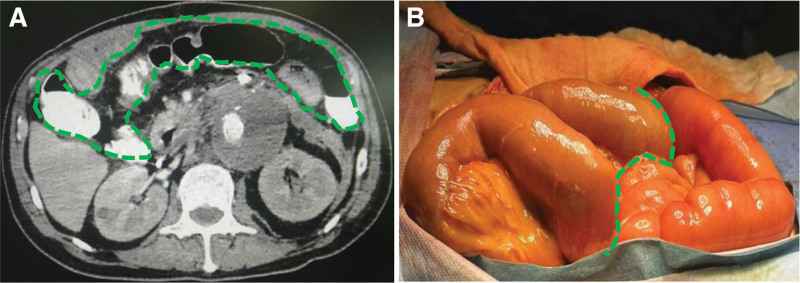
(A) Abdominal enhanced CT scan showing residual contrast in the colon, suggesting possible colonic ischemia. (B) Intraoperative findings showing extensive adhesion of the abdominal intestinal wall. The small intestine appears red and fresh on the right, while the colon is light gray, with no obvious signs of necrosis on the left.

Emergency decompressive laparotomy revealed severely dilated small bowel loops and patchy ischemia of the descending and sigmoid colon. Extensive adhesiolysis was performed, and a diverting loop ileostomy was constructed. Intraoperative assessment showed viable small bowel peristalsis but reduced perfusion evidenced by sigmoid colon pallor and absent mesenteric pulsation (Fig. 2A and B). To enhance the surgical outcome and maintain continuous postoperative decompression, ABTHERA™ negative pressure wound therapy was used, with close monitoring of IAP.

**Figure 3.**
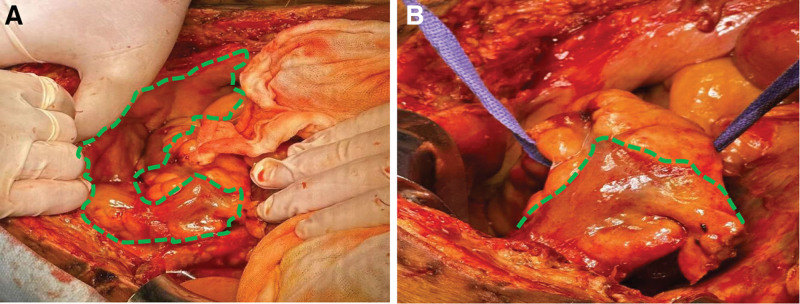
(A) Open intra-abdominal view shows a stiff, bluish-purple colon within green dashed line, indicating ischemia. (B) The anastomosis between the small intestine and the lower rectum after a definitive total colectomy with upper rectal resection. The green dashed line indicates the site of the anastomosis.

Although the postoperative management partially alleviated the IAP, which fluctuated between 20 mm Hg, it was difficult to return to normal levels. Initially, the patient showed signs of improvement, including transient increased urine output, reduced ventilator demand, and stabilization of liver enzymes. However, a week later, the patient developed intermittent fresh melena and hemodynamic instability (Hb 54 g/L). Colonoscopy revealed mucosal sloughing and scattered ulcerations, further exacerbating the abdominal pressure. This resulted in the recurrence of refractory ACS. The prolonged elevated IAP led to ischemia of the colon, which in turn worsened the abdominal distension. A multidisciplinary consultation involving ICU, surgery, anesthesia, and nutrition specialists led to a hypothesis: the colon had been in a state of chronic ischemia for a week, now with scattered ulcers present. Meanwhile, the blood supply to the small intestine remained temporarily stable. Given the sustained high IAP, rather than waiting for irreversible multi-organ failure, we considered abandoning the colon as a pragmaticsolution. Its removal can significantly relieve intra-abdominal pressure and eliminate the source of gastrointestinalbleeding. Furthermore, preserving the small intestine does not significantly impact future enteral nutrient absorption. The patient’s family unconditionally supported this trial and was willing to assume the associated risks. Exploratory laparotomy revealed that the blood supply to the small intestine had not changed since a week ago. A definitive total colectomy with upper rectal resection was then performed. Gross examination confirmed pan-colonic transmural necrosis with serosal discoloration and loss of mural integrity (Fig. 3A and B).

## 7. Follow-up and outcome

Following definitive abdominal closure, the patient demonstrated progressive multi-organ recovery. Diuresis resumed within 48 hours, with serum creatinine stabilizing at baseline (0.8 mg/dL) by postoperative day 7. Concurrently, hepatic transaminases normalized (AST/ALT < 40 U/L), and mechanical ventilation was weaned on day 10. The tracheostomy was decannulated on day 14 after successful swallow evaluation. Enteral nutrition advanced to goal caloric intake (25 kcal/kg/day) via percutaneous jejunostomy without intolerance. After 81 days of hospitalization, the patient was discharged ambulating independently > 100 meters (verified by 6-minute walk test) with a healed midline incision and restored renal/hepatic function. At the 1-month outpatient follow-up, he reported normal oral intake and intermittent loose stools (managed effectively with loperamide 2 mg PRN and fiber supplementation), with no recurrence of gastrointestinal bleeding, organ dysfunction, or wound complications.

## 8. Discussion

Ruptured abdominal aortic aneurysm (RAAA) remains one of the most catastrophic vascular emergencies, with operative mortality rates exceeding 40% to 50% despite significant advancements in surgical techniques.^[1]^ Based on clinical trends in the United States, Endovascular Aneurysm Repair (EVAR) has become the preferred treatment for many cases of AAA, particularly in acute RAAA. In fact, elective EVAR has increased by 0.2 per year, while elective open repair (OAR) has decreased. EVAR is gradually replacing traditional OAR, but it is not suitable for all patients, especially in complex RAAA cases, where a combination of approaches is still required to ensure patient stability.^[3]^ In this case, REBOA was employed, with femoral access used to stabilize circulatory parameters. After initial hemorrhage control, further management was performed through a midline laparotomy and resection of the aneurysmal segment with interposition of a synthetic Dacron graft. This surgical strategy combined the advantages of both endovascular and open surgery, enabling rapid stabilization of the patient’s hemodynamics and swift management of the ruptured aneurysm.

Prompt hemorrhage control is the cornerstone of management, as hypotension and metabolic acidosis—key indicators of severe hemorrhage—are strongly associated with the early onset of MODS postoperatively, worsened by ischemia-reperfusion injury.^[4]^ The Society for Vascular Surgery recommends a door-to-intervention time of <90 minutes in such patients, as early intervention can significantly improve survival outcomes.^[4]^ However, the completion of such early surgical intervention does not signify an easier subsequent management. Ersryd et al^[5]^ identified key risk factors for the development of ACS after endovascular repair of RAAA, including a preoperative BP < 70 mm Hg, the use of an aortic occlusion balloon, and more than 5 intraoperative pRBC unit transfusions. However, Hörer et al^[6]^ found that the use of an aortic occlusion balloon was not independently associated with ACS development. Despite these differing findings, both studies emphasize the critical role of hemodynamic instability and extensive fluid resuscitation as significant contributors to ACS risk following RAAA repair. Our patient’s ACS inevitably aligns with several factors above, including massive transfusion (>6 units) and aortic occlusion balloon. Prolonged intra-abdominal pressure (IAP > 24 mmHg), combined with gastrointestinal dysmotility and reduced pulmonary compliance, evoked a rapid onset of multiorgan complications within 24 hours, and several literatures consistently highlight the role of intra-abdominal hypertension in exacerbating organ failure by diminishing visceral perfusion, particularly in the kidneys and gastrointestinal tract.^[7–9]^ Therefore, despite advanced therapies like continuous renal replacement therapy and molecular adsorbent recirculating system, rapid renal and hepatic dysfunction developed as long as the issue of intra-abdominal hypertension remained unresolved.

Elevated intra-abdominal pressure is known to impair colonic perfusion, and delayed decompression significantly increases the risk of transmural necrosis.^[10]^ In other words, the progression of colonic ischemia and necrosis further exacerbated the elevated intra-abdominal pressure, creating a vicious cycle. The study by Ilic et al^[11]^ identified pre-, intra-, and postoperative risk factors associated with transmural colonic ischemia (CI) following open repair (OR) of RAAA. In a cohort of 89 patients, 14 (15.73%) developed CI. The study found that patients with CI had longer durations of hypotension (42.86 ± 35.82 vs 24.13 ± 23.48 minutes, *P* = .021), more frequent significant hypotension (54.54% vs 14.66%, *P* = .024), and higher mortality (78.57% vs 29.33%, *P* = .001). Risk factors for CI included age over 75, prolonged hypotension, organ injury, abdominal compartment syndrome, and elevated potassium levels. The study concludes that these factors could guide the timely diagnosis and management of colonic ischemia in RAAA patients. While this intervention carries risks, including delayed fascial closure and intestinal ischemia, the 2024 guidelines by Wanhainen et al^[12]^ still stress the importance of early decompressive laparotomy in managing ACS, especially in complex vascular cases, and support the use of negative pressure wound therapy (NPWT) systems, such as ABTHERA™ used in our case, to enhance fluid drainage, reduce fascial tension, and may improve visceral perfusion.^[13]^ When both of the above methods proved ineffective, the decision was made to perform a total colectomy with upper rectal resection, which immediately contributed to breaking this seemingly insurmountable situation. This decision was made through a collaborative multidisciplinary approach, with the unwavering trust and support of the patient’s family. Based on this successful experience, when small bowel function is preserved, could total colectomy be considered an effective and routine treatment option in similar critical scenarios, extending beyond just this specific case? Clearly, further academic discussion, accumulation of additional case studies, and careful ethical considerations would be necessary to evaluate its broader applicability.

Recent studies by Yunus et al^[10]^ emphasize the value of a multidisciplinary, protocolized approach to RAAA management, which is critical for improving outcomes in complex cases like ours. After the multidisciplinary protocol was implemented, mortality rates decreased significantly. In the endovascular repair group, mortality dropped from 46.2% to 20.0% (*P* = .048), and in the open repair group, it fell from 53.3% to 27.3% (*P* = .018). Complication rates also decreased, with a reduction in all-cause morbidity in EVAR patients from 65.4% to 44.0% (*P* = .050) and a significant drop in renal complications from 15.4% to 0.0% (*P* = .036). Bowel ischemia in open repair patients decreased from 26.7% to 0.0% (*P* = .035). Additionally, time to incision and total procedure time were significantly reduced. These improvements indicate that the protocol enhanced patient outcomes and surgical efficiency. Menges et al^[14]^ highlight the value of simulation-based training in enhancing team performance and minimizing intraoperative errors, further supporting its role in improving outcomes in high-stakes surgeries. Similarly, the Swissvasc report^[15]^ emphasizes the importance of centralizing RAAA treatment in high-volume centers, noting that specialized care reduces perioperative complications and improves outcomes in complex surgeries like RAAA repair. We validate the importance of a structured, multi-phase damage control approach in managing RAAA complicated by MODS and ACS through this case, which provides critical insights into integrating modern therapeutic interventions into the management protocol for such complex conditions. We hope to contribute to the ongoing refinement of clinical practice and the development of effective management strategies for RAAA patients with evolving complications.

## 9. Conclusions

This case underscores the challenges in managing RAAA complicated by ACS. Despite initial improvement, persistent elevated intra-abdominal pressure led to ischemia and necrosis of the colon. After multidisciplinary consultation, a total colectomy with upper rectal resection was performed, alleviating intra-abdominal pressure and addressing gastrointestinal bleeding. This intervention, while preserving small bowel function, highlights the potential of total colectomy in such critical scenarios. A protocol-driven, multidisciplinary approach is essential in optimizing outcomes for RAAA patients with complex complications, though further discussion and case studies are needed to assess its broader applicability.

## Acknowledgments

The authors declare that there is no conflict of interest regarding the publication of this manuscript.

## Author contributions

**Writing – original draft:** Yangyang Jiang.

**Writing – review & editing:** Jindi Ni, Lijing Jiang.

**Data curation:** Lu Zhang.

**Supervision:** Xiang Li.
